# Diurnal fluctuating temperature and larval resource level interact to influence the life history and behaviour of disease-transmitting mosquitoes

**DOI:** 10.1186/s13071-026-07313-4

**Published:** 2026-02-21

**Authors:** Juliah Wanjiru Jacob, Laura Grenville-Briggs, Merid Negash Getahun

**Affiliations:** 1 Department of Plant Protection Biology, Swedish University of Agricultural Sciences, Alnarp, Sweden; 2 International Center of Insect Physiology and Ecology, Nairobi, Kenya

**Keywords:** Carry, Over effects, Temperature, And resource, Dependent life history traits, Mosquitoes

## Abstract

**Background:**

The global rise in temperature has seen a geographical range increase in mosquito populations and disease transmission. Temperature affects life history traits of mosquitoes, and hence, population dynamics and vectorial capacity. In addition, food resource abundance, required for biomass and somatic energy during mosquito larval development is dependent on temperature. How the interaction between temperature and food resources affects the life history traits of aquatic stages, and subsequent carry-over effects to the adult stage, under simulated natural conditions, remains underexplored.

**Methods:**

A comparative assessment of the interactive effect of diurnal fluctuating temperature and resource level during larval development on life history traits of the yellow fever mosquito, *Aedes aegypti*, and three malaria vectors, *Anopheles stephensi*, *Anopheles coluzzii* and *Anopheles arabiensis* was conducted. Moreover, carry-over effects on teneral adults including, metabolic reserves and propensity to feed were evaluated on the four species under similar abiotic conditions. A total of 2700 larvae of each species were reared under three fluctuating temperature regimes, and maintained on different resource levels. A mixed-effects Cox regression model was used to determine effects of the two environmental factors on the time to adult emergence, and adult survival. Generalised linear mixed-effect model with a binomial error structure was used to elucidate effects of abiotic stress on feeding, whereas linear-mixed effects analysis of variance, was used to estimate the effects of temperature and resource level on adult size and metabolic reserves. Aligned Rank Transform analysis of variance was used to determine effects of abiotic stress on level of feeding. Correlation between size and survival of starved adults was determined by multivariate analysis using Spearman’s rank correlation and linear regression.

**Results:**

Time to adult emergence shortened with increasing temperature and resource level. Accelerated adult emergence was associated with reduced adult size and survival at high temperature, in a resource-dependent manner. Metabolic macronutrient reserves carried over into teneral adults were differentially regulated by temperature and larval resource level, in a species dependent manner. Teneral females engaged in feeding on honey or blood depending on the two abiotic stressors, and species.

**Conclusions:**

Temperature and resource level during larval development differentially affects life history traits of disease-transmitting mosquitoes, which have ramifications on population size, as well as disease transmission dynamics.

**Graphical Abstract:**

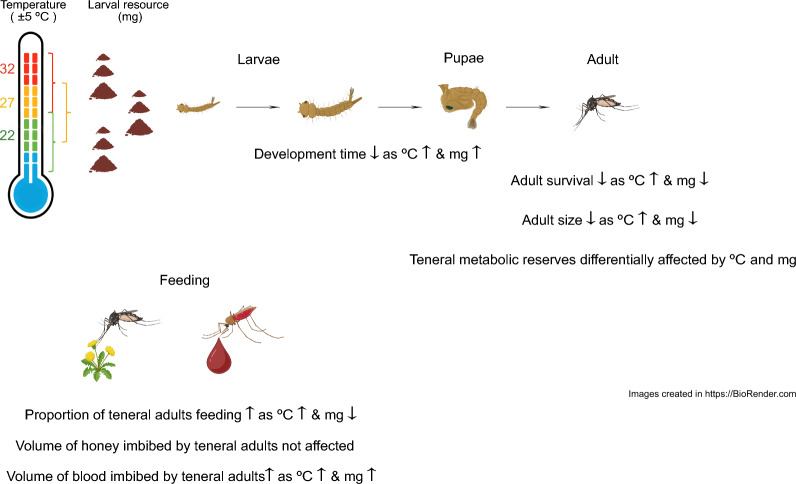

**Supplementary Information:**

The online version contains supplementary material available at 10.1186/s13071-026-07313-4.

## Background

Vector-borne diseases are a key global health challenge, exacerbated by climate change as a consequence of anthropogenic activities [1–3]. As ectotherms, the life history traits, physiology and behaviour of mosquitoes are sensitive to changes in temperature, which ultimately determine their geographical distribution and vectorial capacity [4–15]. However, previous studies do not fully reflect the effect of temperature on mosquito life history and physiology, as most were conducted under constant temperatures. Therefore, to better predict disease spread, it is important to study how varying temperatures will affect vector population dynamics in the face of climate change [16, 17]. Temperature conditions may, likewise, affect larval food availability in breeding habitats, including, *e.g*., microorganisms and plant detritus [18, 32–34] by regulating the abundance of ectothermic microorganisms [63]. Subsequently, this will indirectly regulate fitness parameters of mosquito larvae and adults [19]. In light of global warming, understanding the response of mosquitoes, and their associated food sources, to fluctuating temperatures may be critical for generating accurate future geographical distribution models [18], as well as estimating vector availability and human biting rate in vectorial capacity models of malaria and dengue transmission [13, 20].

Available data on the effect of diurnal temperature fluctuation on mosquito life history traits emphasise that this effect is non-linear, unimodal and restricted within temperature safety margins, defined as critical temperature maxima and minima within which optimal fitness is displayed [13, 14, 16, 20]. For instance, vital life history traits for malaria and dengue vectors are optimal within 23 °C—29 °C, below or above which the development rate, survival, fecundity, biting rate and pathogen transmission decline [13, 16, 20]. Mosquito temperature safety margins are dependent on habitat temperature ranges, with safety margins being more constricted for tropical species and during summer for temperate species, with the latter broadening the margin during cooler months [21, 22]. Moreover, temperature fluctuations within an ecological zone may influence mosquito life history traits. For instance, the aquatic stages of the malaria vector *Anopheles arabiensis* is more tolerant to higher temperatures compared with *Anopheles funestus* and the sympatric species, *Anopheles gambiae *sensu stricto [23]. At high average daily temperatures, saltatory diurnal temperature changes within the critical temperature range reduced the development rate, survival and increased the length of the gonotrophic cycle, and vectorial capacity of the malaria vectors, *An. gambiae s. s.* and *Anopheles stephensi*, as well as the dengue vector, *Aedes aegypti* [24–27].

Studies examining the effects of temperature on mosquito physiology and behaviour are limited. However, existing reports suggest that higher constant daytime temperatures are negatively correlated with metabolic reserves, adult size and blood-feeding behaviour of mosquitoes [8, 28]. As such, cross-species analysis of the effects of fluctuating temperature on the life history parameters of larvae and the ensuing carry-over effects, *i.e*., the irreversible larval fitness advantages transferred to the adult stage [29] should be elucidated. This may provide a much-needed holistic understanding of the differential impact of global warming on major malaria and dengue vectors.

Food availability affects mosquito fitness because metabolic rate scales with temperature [64]. Therefore, the teneral adult somatic energy requirements across different temperature gradients is limited by larval food abundance [19, 30, 31]. Larvae from nutrient-rich habitats, irrespective of temperature stress, have a ‘silver spoon’ advantage, *i.e.,* a better start [29], and produce adults with increased fitness, immunity and metabolic reserves, while those from resource-poor habitats have reduced fitness and reserves [4, 19, 29, 35–40].

Under natural conditions, how the interaction between temperature and food resources shape fitness and, by extension, the vectorial capacity of mosquitoes, remains elusive. In this study, the interactive effect of dynamic temperature and varying resource levels, on mosquito development time, and the carry-over effects on adult life history traits, physiology and behaviour were evaluated. To gain insight into the variability of this effect, a comparative analysis was made among *Ae. aegypti* and three major malaria vectors, *An. stephensi*, *Anopheles coluzzii* and *An. arabiensis*. Our findings provide insight into how the interaction of environmental factors may shape the fitness, and potentially the vectorial capacity, of mosquito vectors in view of climate change.

## Methods

### General colony maintenance

Long standing laboratory colonies of *Aedes aegypti* (Rockefeller), *An. stephensi* (type-form, SDA-500), *An. coluzzii* (G3) and *An. arabiensis* (Dongola) were reared at a 12 h: 12 h light: dark photoperiod, 25 ± 2 °C and 65 ± 5% relative humidity. Adults were provided 10% sucrose *ad libitum*, and mature females offered defibrinated sheep blood (Håtunalab AB, Bro, Sweden) via membrane feeding (Hemotek Ltd, Blackburn, UK). Ovicups (30 ml; Nolato Hertila AB, Åstorp, Sweden) lined with filter paper (90 mm; Whatman®, Thermo Fischer Scientific, Gothenburg, Sweden) and half filled with distilled water were provided as an oviposition substrate to gravid females for egg deposition. Eggs were hatched in plastic trays (7 cm × 18 cm × 20 cm), containing 1 L of distilled water. Upon emergence, larvae were maintained on ca ~0.75 mg larva day^−1^ of Tetramin® fish food (Tetra GmbH, Melle, Germany) until pupation. Pupae were collected into ovicups (30 ml; Nolato Hertila AB), which were placed in Bugdorm cages (30 cm × 30 cm × 30 cm; MegaView Science Co., Ltd, Taichung, Taiwan), and emerging adults maintained as described above.

### Experimental design

To evaluate how ambient temperature and resource restriction interactively affect fitness and feeding behaviour of disease vectors, a 3 × 3 × 4 factorial design, including three resource regimes, three fluctuating diurnal temperature ranges, and four mosquito species, was used (Additional file 1: Supplementary Fig. S1). Experimental chambers (IPP750ecoplus; Memmert GmbH, Büchenbach, Germany) were programmed to either of the three temperature regimes, 22 ± 5 °C, 27 ± 5 °C and 32 ± 5 °C with a gradual increase and decrease in temperature during photophase and scotophase, respectively, reflecting the natural change on the basis of meteorological data (AccuWeather; Kilifi, Kenya). The low temperature was fluctuated at a range of 17–27 °C around the mean of 22 °C, intermediate temperature at 22–32 °C around a mean of 27 °C and high temperature at 27–37 °C around a mean of 32 °C (Additional file 1: Supplementary Fig. S2). The temperature regimes were selected on the basis of the geographical range distributions of the four mosquito species [41–44], reflecting both cooler and warmer climates. A photoperiod of 12 h: 12 h light: dark was used, and a 30 min gradual transition, at 30% light intensity, between photoperiods was adopted to simulate sunrise and sunset. Relative humidity in the experimental chambers was not regulated and it ranged from 90 to 100%. Data loggers (Gemini Data Loggers Ltd, Chichester, UK) were used to monitor temperature and humidity in the climatic chambers throughout the experimental period.

For *Ae. aegypti*, a high resource level of Tetramin® fish food (1 mg larva^−1^ day^−1^) was selected on the basis of previous studies showing this to be the amount of food supporting the highest larval survival [19]. To measure the effect of resource limitation, the high resource level was scaled down by a factor of 10^0.5^ and 10^1^ to obtain a ‘mid’- and low-resource level of 0.32 mg larva^−1^ day^−1^ and 0.1 mg larva^−1^ day^−1^, respectively. This resource regime was equally adopted for the maintenance of *An. stephensi* since the two species are known to share breeding habitats [45]. For *An. coluzzii* and *An. arabiensis*, a high resource level of 0.75 mg larva^−1^ day^−1^ was adopted from amounts used in routine laboratory colony maintenance of these species [46]. This amount was scaled down using similar factors as in *Ae. aegypti* to obtain a mid- and low-resource level of 0.24 mg larva^−1^ day^−1^and 0.075 mg larva^−1^ day^−1^, respectively.

To begin the experiments, eggs obtained from laboratory colonies of the four species were hatched in distilled water that was previously incubated in the three experimental chambers for 24 h. Cohorts of 50 first-instar larvae were transferred into 1 L distilled water in plastic trays (7 cm × 18 cm × 20 cm). For each species, this was replicated six times resulting in 6 × 3 temperature ranges × 3 resource levels = 54 experimental units, and a total number of 10,800 mosquitoes. Owing to space limitation in experimental chambers, the effect of abiotic stress on all species was not evaluated at the same time. Therefore, the effect each resource level on fitness traits across temperature regimes was evaluated per species, at a time.

### Fitness traits

Daily larval mortality was monitored for each treatment, and the amount of larval food adjusted so as to maintain a constant resource supply (mg larva^−1^ day^−1^) throughout the experiment. To manage resource quantity and prevent detrimental microbial growth, larval trays were cleaned and the water changed daily. For each replicate per treatment, pupae were collected and pulled into ovicups as per the date of pupation. The ovicups were placed in Bugdorm cages (MegaView Science) and, upon emergence, the adults were maintained on distilled water throughout their life span in the experimental chambers. The development time of mosquitoes surviving to adult stage was determined as the time from egg hatching to adult emergence. Adults were grouped as per the date of emergence to account for their survival. Survival of the starved adults was used to evaluate whether metabolic reserves carried over from immature stages contributed to resilience of the four species when subjected to different temperature and larval resource regimes. The wing length (mm) from five females per replicate in each treatment was used as a proxy for adult size [47]. Length of the left wing was measured from the distal end of the alula up to the tip, excluding the fringe, using a dissecting microscope fitted with an ocular micrometer. To establish whether the survival of starved adults was dependent on size, the correlation between average size and survival, *i.e.*, time to death (in days), for each temperature and resource level, was determined.

### Metabolic macronutrient energy reserves

Metabolic reserves were estimated from ten < 6 h post-emergence teneral mosquitoes, reared as described above, across the different temperature regimes and resource levels. Total soluble protein, total glycogen and carbohydrate, as well as lipid amounts were fractionated from individual insects [48]. Proteins forms the structural component, while glycogen and lipids are the major storage forms of energy. Glycogen represents the first choice for storage and use, as these are readily converted to carbohydrates, and carbohydrates are the readily available metabolic fuel for insects [59]. The amount of metabolic reserves in each individual mosquito was normalized by the respective average wing size, for each temperature and resource regime [52].

### Analysis of total soluble proteins

Briefly, individual mosquitoes (*n* = 10) were placed in 2 ml microfuge tubes, macerated in 180 µl protein lysis buffer, agitated for 1 min and then centrifuged at 6700 RCF for 5 min. An aliquot of the supernatant (10 µl) was transferred to a separate microfuge tube, containing Coomassie blue dye (250 µl, Bio-Rad Laboratories AB, Solna, Sweden), vortexed for 2 min, incubated at room temperature for 17.5 ± 2.5 min and the absorbance measured at 595 nm (Multiskan FC, Thermo Fischer Scientific, Sweden). Protein content was determined using a bovine serum albumin (BSA) (Bio-Rad Laboratories AB) standard curve [49, 75].

### Analysis of total carbohydrates and glycogen

To the remaining fraction of the sample (170 µl left after an aliquot of 10 µl was used for protein analysis), 20% sodium sulphate (20 µl, Merck Life Science AB, Solna, Sweden) and 1:1 methanol: chloroform (1.5 ml, both 99%, Merck Life Science AB) mixture were added and centrifuged at 6700 RCF for 5 min. The supernatant was transferred to a new microfuge tube. Total carbohydrates, in the methanol fraction, were separated from the lipid fraction, dissolved in chloroform, by the addition of 500 µl distilled water to the supernatant and then centrifuged at 6700 RCF for 5 min. Total carbohydrates in methanol fraction, and the pellet containing total glycogen were analysed using the hot anthrone reaction, and the absorbance measured at 620 nm (Multiskan FC, Thermo Fisher Scientific) [50, 75]. Total carbohydrates includes simple sugars, glucose, fructose, sucrose, oligosaccharides and all polysaccharides (which include glycogen and starch) present in the mosquitoes tissue [59]. Glycogen analysis, is specific to the amount of glycogen, a large, branched polysaccharide made entirely of glucose units and serving as a major energy storage molecule in insects [59]. By measuring both total carbohydrates and glycogen, it is possible to distinguish between readily available energy sources and long-term energy reserves in mosquito tissues [50, 59]. Amounts of total carbohydrates and glycogen were estimated from a D-glucose (99.5%, Merck Life Science AB) standard curve.

### Analysis of total lipids

Lipids dissolved in the chloroform fraction were analysed in vanillin reagent (99%, Merck Life Science AB), and the absorbance measured at 520 nm (Multiskan FC, Thermo Fisher Scientific) [51, 75]. Lipid content in individual mosquitoes was quantified using olive oil standard curve [51, 75].

### Feeding assay and volumetric analysis

To evaluate how temperature and resource level during larval development affects adult feeding behaviour, newly emerged females were offered either a carbohydrate- (bee honey, ICA AB, Solna, Sweden; diluted in distilled water to 60% vol/vol) or protein-rich meal (sheep blood). *Aedes aegypti* were fed during photophase (Zeitgeber time, ZT, 2–5), whereas the anophelines were fed during scotophase (ZT 13–16), analogous to their diel-activity period [53, 54]. Mosquitoes were provided either 60% honey, mixed with 1 mg ml^−1^ xylene cyanol (Merck Life Science AB), from 0.2 ml PCR strip tube caps (VWR, Stockholm, Sweden) for 3 h, or blood via membrane feeding (Hemotek Ltd) for 1 h. Blue colouration of the abdomen was used to score honey-fed from unfed insects. Ingested volumes of honey and blood from fed females were quantified by spectrophotometry, as previously described by Dawit et al. [55] and Briegel et al. [56], respectively. The volumes obtained were adjusted by the average size of females, from the respective treatments (Fig. 2). For each temperature and resource level, > 30 females were used in feeding assays, and in cases with low feeding propensity the sample size was increased so as to obtain insects for volumetric analysis.

### Statistical analysis

Mixed-effects Cox regression analysis was used to estimate the effects of temperature, resource level and their interaction on the time to adult emergence. Kaplan–Meier survival curves, plotted using the survminer package, were used to visualise differences in adult survival across different experimental treatments. The effects of temperature and resource level, and their interaction on survival were determined using the mixed-effect Cox regression model [75]. Linear-mixed effects analysis of variance, using Kenward–Roger degrees of freedom approximation, was used to estimate the effects of temperature, resource level and their interaction on adult size and metabolic reserves. Pairwise comparison of emmeans was used to decipher significant interactions of the two abiotic factors across different treatments, and *P*-values were corrected by the Tukey method [57]. These analysis were conducted using the lmerTest and coxme packages [86]. Effects of temperature and resource level on feeding behaviour of newly emerged females was evaluated using the generalised linear mixed-effects model with a binomial error structure [75]. Significant interactions of the two experimental variables on the proportion of adults feeding were estimated by pairwise comparison of emmeans using Tukey method adjusted *P*-values [57]. The Aligned Rank Transform (ART) analysis of variance was used to elucidate effects of temperature, resource level and their interaction on the volume of honey and blood ingested by teneral females. The non-parametric ART analysis of variance was selected due to heteroscedasticity of the volumetric data following normality test with Shapiro–Wilcoxon analysis [65, 58]. This analysis was performed using the art function in ARTool package. Post hoc analysis of significant interactions of temperature and resource level on fitness traits was evaluated using the art.con function, and Tukey method adjusted *P*-values. In all models, replicate was treated as a random effect, and date was randomised in time to adult emergence. Temperature and resource level were modelled as fixed effects. Correlations between size and survival were determined using the non-parametric multivariate analysis of temperature, resources level, size and survival by Spearman’s rank correlation. Moreover, differences in the effect of size on survival across different temperature regimes were analysed using linear regression by comparing slopes and *y*-intercepts and correcting for multiple comparisons using Tukey-adjusted *P*-values. Spearman’s rank correlation and linear regression were conducted using the cor.test and lm functions, respectively [86]. All analytical tests were carried out in R (version 4.3.1, R core development team, 2023).

## Results:interaction of temperature and resource level impacts life history traits of four mosquito species

### Time to adult emergence

**Fig. 1:**
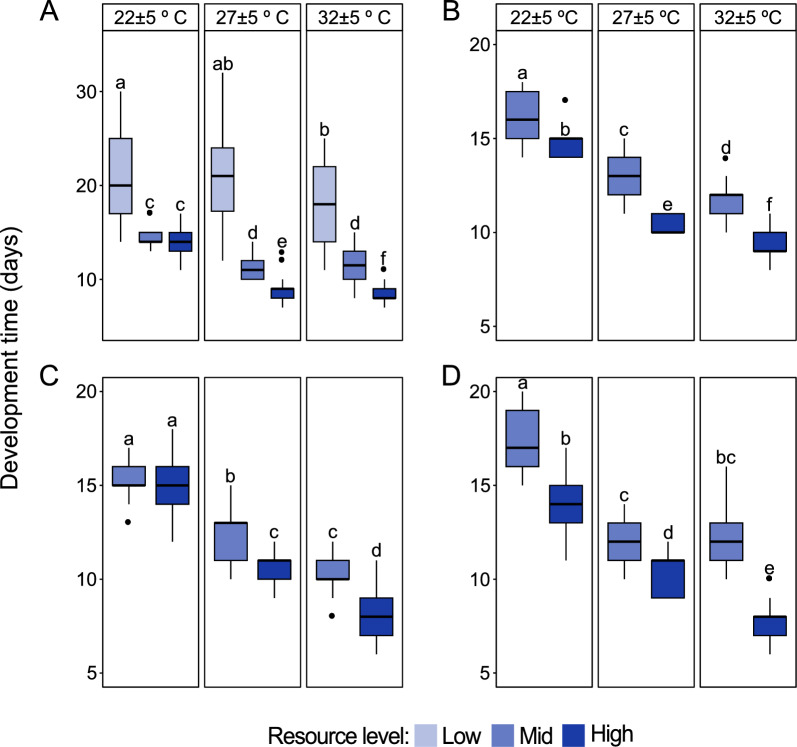
Temperature and resource level interact to influence the development time of mosquitoes. Development time from egg hatching to adult emergence of female **A** *Ae. aegypti* (22 ± 5 °C: high resource *n* = 112; mid resource *n* = 105; low resource *n* = 87; 27 ± 5 °C: high resource *n* = 93; mid resource *n* = 114; low resource *n* = 85; 32 ± 5 °C: high resource *n* = 107; mid resource *n* = 94; low resource *n* = 70), **B** *An. stephensi* (22 ± 5 °C: high resource *n* = 81; mid resource *n* = 38; 27 ± 5 °C: high resource *n* = 115; mid resource *n* = 107; 32 ± 5 °C: high resource *n* = 111; mid resource *n* = 75) **C** *An. coluzzii* (22 ± 5 °C: high resource *n* = 92; mid resource *n* = 81; 27 ± 5 °C: high resource *n* = 103; mid resource *n* = 70; 32 ± 5 °C: high resource *n* = 77; mid resource *n* = 49) and **D** *An. arabiensis* (22 ± 5 °C: high resource *n* = 104; mid resource *n* = 81; 27 ± 5 °C: high resource *n* = 88; mid resource *n* = 69; 32 ± 5 °C: high resource *n* = 62; mid resource *n* = 22) in response to diurnal fluctuating temperature and resource level. Lowercase letters, from pair-wise comparisons of emmeans, indicate significant interactions of temperature and resource level on the development time (*P* < 0.05; mixed-effect Cox regression analysis). At low larval resource level *Anopheles* species did not pupate, and hence no data on development time at this resource level

The time to adult emergence of all the four species decreased with an increase in temperature (*Ae. aegypti*: df = 2, *χ*^2^ = 227.70, *P* < 0.001; *An. stephensi*: df = 2,* χ*^2^ = 296.15, *P* < 0.001; *An. coluzzii*: df = 2, *χ*^2^ = 228.39, *P* < 0.001; *An. arabiensis*: df = 2, *χ*^2^ = 183.99, *P* < 0.001) and an increase in resource level (*Ae. aegypti*: df = 2, *χ*^2^ = 698.82, *P* < 0.001; *An. stephensi*: df = 1, *χ*^2^ = 140.46, *P* < 0.001; *An. coluzzii*: df = 1, *χ*^2^ = 45.55, *P* < 0.001; *An. arabiensis*: df = 1, *χ*^2^ = 90.21, *P* < 0.001) (Fig. 1A–D). Moreover, high larval resource level shortened the time to adult emergence of *Ae. aegypti* (Fig. 1A) and *An. coluzzii* (Fig. 1C) at high temperature (*Ae. aegypti*: df = 4, *χ*^2^ = 204.89, *P* < 0.001; *An. coluzzii*: df = 2, *χ*^2^ = 21.96, *P* < 0.001), while for *An. stephensi* (Fig. 1B) and *An. arabiensis* (Fig. 1D) this was observed across all temperature regimes (*An. stephensi*: df = 2, *χ*^2^ = 20.88, *P* < 0.001; *An. arabiensis*: df = 2, *χ*^2^ = 27.31, *P* < 0.001). At low larval resource level, *Anopheles* species larvae did not transition to pupae.

### Adult size

**Fig. 2:**
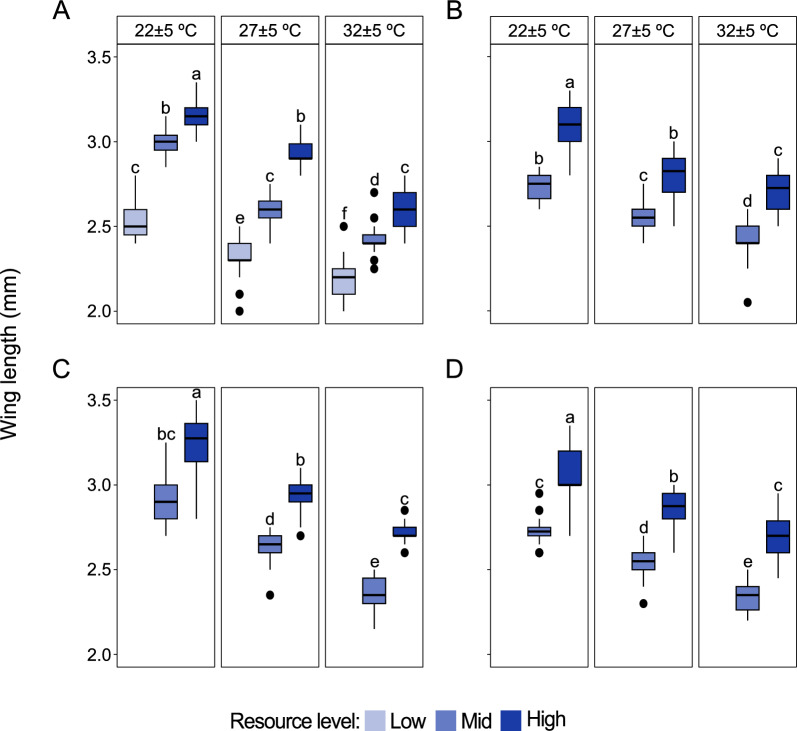
Temperature and resource level interact to influence the adult size of mosquitoes. The wing length of female **A** *Aedes aegypti*, **B** *Anopheles stephensi*, **C** *Anopheles coluzzii* and **D** *Anopheles arabiensis* was used as a proxy for adult size. For all species, *n* = 30 in each treatment regime. Lowercase letters, from pair-wise comparisons of emmeans, indicate significant interactions of temperature and resource level on the size for each species. Bars followed by different letters within the same box (between resource levels and same temperature) and bars across boxes (between temperature and same resource) are statistically different when represented by different letters (*P* < 0.05; Mixed-effect ANOVA). At low larval resource level *Anopheles* species did not pupate, and hence no data on size at this resource level

The size of all the four species decreased with an increase in temperature (*Ae. aegypti*: F_2,261_ = 581.14, *P* < 0.001; *An. stephensi*: F_2,174_ = 301.41, *P* < 0.001; *An. coluzzii*: F_2,175_ = 151.76, *P* < 0.001; *An. arabiensis*: F_2,158_ = 92.19, *P* < 0.001) and increased with an increase in resource level (*Ae. aegypti*: F_2,261_ = 730.55, *P* < 0.001; *An. stephensi*: F_1,174_ = 346.22, *P* < 0.001; *An. coluzzii*: F_1,175_ = 323.49, *P* < 0.001; *An. arabiensis*: F_1,158_ = 312.76, *P* < 0.001) (Fig. 2A–D). Across all temperature regimes, *Ae. aegypti* (Fig. 2A) and *An. coluzzii* (Fig. 2C) females were bigger in size when maintained on a high resource level during larval development (*Ae. aegypti*:F_4,261_ = 14.46, *P* < 0.001; *An. coluzzii*: F_2,175_ = 3.31, *P* = 0.039). Temperature and resource level did not significantly interact to influence the size of *An. stephensi* (F_2,174_ = 0.19, *P* = 0.83; Fig. 2B) and *An. arabiensis* (F_2,158_ = 0.27, *P* = 0.76; Fig. 2D). When maintained on a low larval resource level, *Anopheles* species larvae did not transition to pupae, and hence no adults.

### Adult survival

**Fig. 3:**
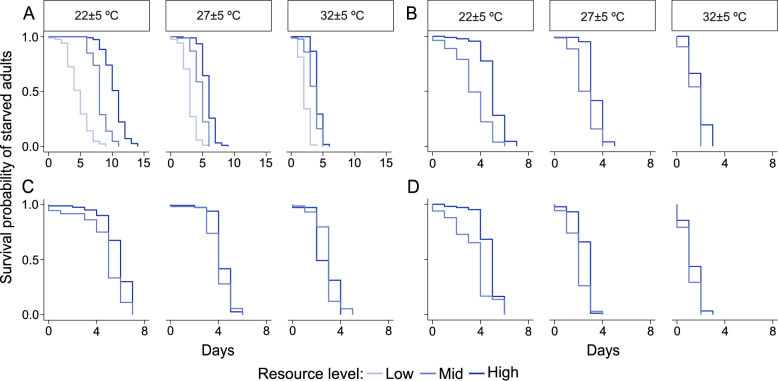
Temperature and resource level interact to influence the adult survival of mosquitoes. Adult survival of female **A** *Ae. aegypti*, (22 ± 5 °C: high resource *n* = 112; mid resource *n* = 105; low resource *n* = 87; 27 ± 5 °C: high resource *n* = 93; mid resource *n* = 114; low resource *n* = 85; 32 ± 5 °C: high resource *n* = 107; mid resource *n* = 94; low resource *n* = 70), **B** *An. stephensi* (22 ± 5 °C: high resource *n* = 81; mid resource *n* = 38; 27 ± 5 °C: high resource *n* = 115; mid resource *n* = 107; 32 ± 5 °C: high resource *n* = 111; mid resource *n* = 75), **C** *An. coluzzii* (22 ± 5 °C: high resource *n* = 92; mid resource *n* = 81; 27 ± 5 °C: high resource *n* = 103; mid resource *n* = 70; 32 ± 5 °C: high resource *n* = 77; mid resource *n* = 49) and **D** *An. arabiensis* (22 ± 5 °C: high resource *n* = 104; mid resource *n* = 81; 27 ± 5 °C: high resource *n* = 88; mid resource *n* = 69; 32 ± 5 °C: high resource *n* = 62; mid resource *n* = 22) in response to diurnal fluctuating temperature and resource level. Significant interactions of temperature and resource level on adult survival are shown in Table 1. At low larval resource level *Anopheles* species did not pupate, and hence no data on survival at this resource level

The survival probability of starved *Ae. aegypti* decreased with an increase in temperature (df = 2, *χ*^2^ = 594.95, *P* < 0.001) and a decrease in resource level (df = 2, *χ*^2^ = 559.70, *P* < 0.001). However, at low temperatures, *Ae. aegypti* survived longer when maintained on a high larval resource level (df = 4, *χ*^2^ = 30.89, *P* < 0.001), a pattern not observed at higher temperatures (Fig. 3A; Table 1). Temperature and resource level did not significantly interact to influence survival of the three starved anopheline species (*An. stephensi*: df = 2, *χ*^2^ = 5.32, *P* = 0.070; *An. coluzzii*: df = 2, *χ*^2^ = 2.62, *P* = 0.27; *An. arabiensis*: df = 2, *χ*^2^ = 1.16, *P* = 0.56; Fig. 3B–D; Table 1), although survival probability of *An. coluzzii* and *An. arabiensis* decreased with an increase in temperature (*An. coluzzii*; df = 2, *χ*^2^ = 242.032, *P* < 0.001; *An. arabiensis*: df = 2 *χ*^2^ = 317.10, *P* < 0.001) and a decrease in resource level (*An. coluzzii*: df = 1, χ^2^ = 45.53, *P* < 0.001; *An. arabiensis*: df = 1, *χ*^2^ = 23.46, *P* < 0.00) (Fig. 3C, D). While high temperature (df = 2, *χ*^2^ = 242.20, *P* < 0.001) decreased the survival probability of *An. stephensi*, resource level (df = 1, *χ*^2^ = 3.42, *P* = 0.06) did not influence the survival of this species (Fig. 3B). There was no adults of *Anopheles* species at low resource level since larvae maintained on this resource regime did not transition to pupae.

**Table 1:** Mixed-effect Cox regression analysis on the interactive effect of temperature and resource level on adult mosquito survival

Species	Temperature (± 5 °C)	Resource level (mg larva^−1^ day^−1^)		
		0.1	0.32	1
*Aedes aegypti*	22	cd	b	a
	27	f	d	c
	32	g	e	e
*Anopheles stephensi*	22	–	a	a
	27	–	b	b
	32	–	c	c

		(mg larva^−1^ day^−1^)		
		0.075	0.24	0.75
*Anopheles arabiensis*	22	–	b	a
	27	–	c	c
	32	–	e	e
*Anopheles coluzzii*	22	–	b	a
	27	–	c	b
	32	–	e	d

Different alphabetical letters indicate significant interaction between temperature and resource level for each species (*P* < 0.05)

### Effect of size on adult survival

Survival of starved adults of all the four species significantly increased with size (*Ae. aegypti*: *ρ* = 0.85, *P* < 0.0001; *An. stephensi*: *ρ* = 0.55, *P* < 0.0001; *An. coluzzii*: *ρ* = 0.73, *P* < 0.0001; *An. arabiensis*: *ρ* = 0.70, *P* < 0.0001), although the relationship between body size and survival decreased with increasing temperature (*Ae. aegypti*: *ρ* = −0.67, *P* < 0.0001; *An. stephensi*: *ρ* = −0.72, *P* = 0001; *An. coluzzii*: *ρ* = −0.70, *P* < 0.0001; *An. arabiensis*: *ρ* = −0.75, *P* < 0.0001;Additional file 1: Supplementary Fig. S3A–D). The effect of size on survival of starved *Ae. aegypti* (F_2,856_ = 72.41, *P* < 0.0001) was higher than that of the *Anopheles* species (*An. stephensi*: F = _2,524_ = 9.1, *P* < 0.0001; *An. arabiensis*: F_2,433_ = 9.28, *P* < 0.0001; *An. coluzzii*: F_2,469_ = 10.91, *P* < 0.0001), but the effect decreased with increasing temperature (Additional file 1: Supplementary Fig. S3A–D; Additional file 2: Supplementary Table S1). The effect of size on survival of starved *Ae. aegypti* and *An. arabiensis* significantly decreased with an increase in temperature up to 27 °C, whereas at 32 °C this effect was low. Conversely, for *An. stephensi* and *An. coluzzii* the size-dependent survival decreased at the highest temperature, 32 °C (Additional file 2: Supplementary Table S1). The effect of size on survival of starved adults of all species significantly increased with an increase in resource level (*Ae. aegypti*: *ρ* = 0.53, *P* < 0.0001; *An. stephensi*: *ρ* = 0.13, *P* = 0036; *An. coluzzii*: *ρ* = 0.32, *P* < 0.0001; *An. arabiensis*: *ρ* = 0.21, *P* < 0.0001), in which larger females emerging from high larval resource regime lived longer compared with smaller females from low-resource level.

### Metabolic reserves

**Fig. 4:**
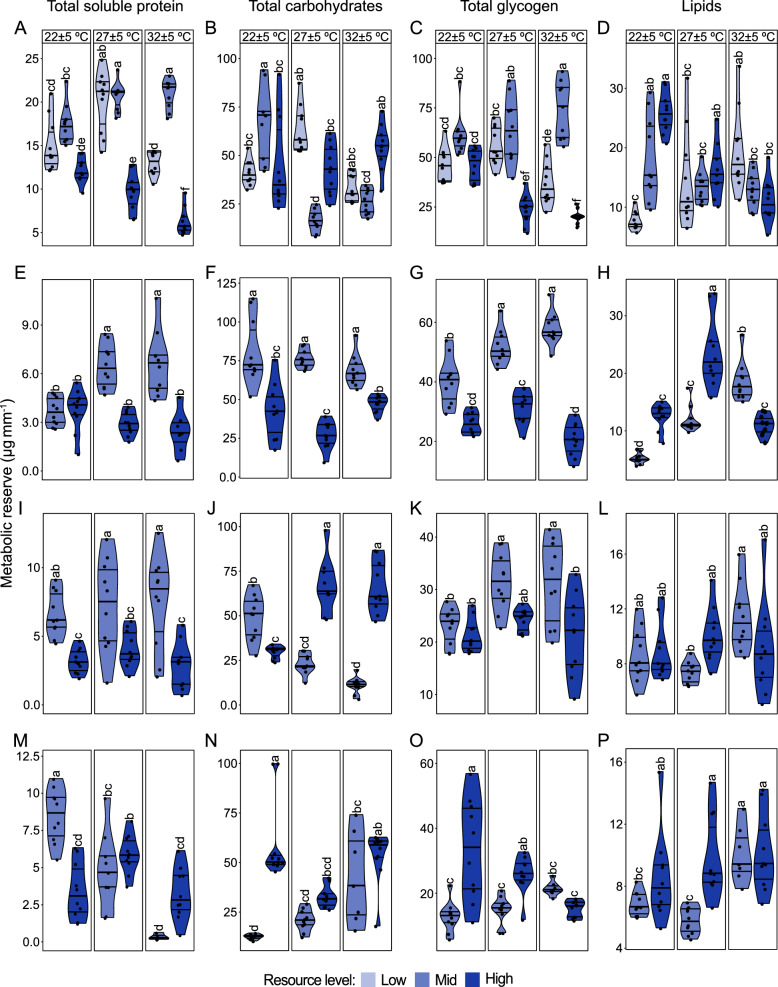
Temperature and resource level interact to influence the teneral metabolic reserves of mosquitoes. Metabolic macronutrients adjusted by mean wing size, in teneral female (**A**-**D**) *Ae. aegypti*, (**E**-**H**) *An. stephensi*, (**I**-**L**) *An. coluzzii* and (**M**-**P**) *An. arabiensis*. For all species, *n* = 10 in each treatment regime. Lowercase letters, from pair-wise comparisons of emmeans, indicate significant interactions of temperature and resource level on the macronutrient for each species. Bars followed by different letters within the same box (between resource levels and same temperature) and bars across boxes (between temperature and same resource) are statistically different when represented by different letters (*P* < 0.05; mixed-effect ANOVA). At low larval resource level *Anopheles* species did not pupate, and hence no data on macronutrients at this resource level

Metabolic reserves carried over from the larval to adult stage of the four species were determined by the interaction of temperature and resource level during aquatic development (Fig. 4A–P). The total soluble protein (Fig. 4A) in teneral *Ae. aegypti* increased with an increase in temperature when females were maintained on mid resource level (F_4,54_ = 18.06, *P* < 0.001). However, at high temperature this macronutrient decreased in insects maintained on a high resource level (Fig. 4A). Total carbohydrates content (Fig. 4B) was high in females reared under low temperature and maintained on a mid resource level, while a decrease in this reserve was observed at intermediate temperature in insects maintained on a similar resource regime (F_4,54_ = 16.71, *P* < 0.001). Moreover, at high temperature, the glycogen content (Fig. 4C) in teneral *Ae. aegypti* decreased in females reared on a high resource level, while this macronutrient increased in females maintained on mid larval resource level (F_4,54_ = 14.50, *P* < 0.001). Total soluble lipid content (Fig. 4D) increased when teneral females were reared under low temperature and maintained on a high- compared with low-larval resource level (F_4,54_ = 11.14, *P* < 0.001).

As temperature increased, the total soluble protein (Fig. 4E), glycogen (Fig. 4G) and lipids (Fig. 4H) increased in teneral *An. stephensi* reared on mid larval resource level (proteins: F_2,26_ = 15.03, *P* < 0.001; glycogen: F_2,27_ = 22.04, *P* < 0.001; lipids: F_2,27_ = 49.40, *P* < 0.001), while at low temperature the lipid content decreased in females maintained on a similar resource level (Fig. 4H). Across all temperature regimes, the total carbohydrates (Fig. 4F) increased in teneral *An. stephensi* maintained on mid resource level during larval development (F_2,54_ = 9.77, *P* < 0.001). Conversely, temperature and resource level did not significantly interact to determine the protein (Fig. 4I) and glycogen (Fig. 4K) content (protein: F_2,27_ = 0.62, *P* = 0.54; glycogen:F_2,27_ = 2.92, *P* = 0.071) in *An. coluzzii*, although across all temperatures, and at high temperature, protein and glycogen, respectively, increased in teneral females maintained on mid- compared with high-resource level during larval development. However, as temperature increased, total carbohydrates (Fig. 4J) increased when teneral *An. coluzzii* were reared on high larval resource level, whereas at low temperature this macronutrient decreased in females maintained on a similar resource level (F_2,27_ = 66.06, *P* < 0.001). Total soluble lipid content (Fig. 4L) increased in teneral females reared under high- compared with intermediate-temperature when larvae were maintained on mid larval resource level (F_2,27_ = 6.27, *P* < 0.01). As temperature decreased, total soluble protein content (Fig. 4M) in female *An arabiensis* increased when reared on mid larval resource level (F_2,26_ = 21.78, *P* < 0.001), whereas total carbohydrates (Fig. 4N) and glycogen (Fig. 4O) increased in teneral females maintained on a high resource level during larval development (carbohydrates: F_2,26_ = 13.86, *P* < 0.001; glycogen: F_2,26_ = 14.58, *P* < 0.001). While at intermediate temperature the total soluble lipids (Fig. 4P) decreased in teneral *An. arabiensis* maintained on mid larval resource level, generally, as temperature increased this macronutrient increased in females reared on mid resource level (F_2,26_ = 4.086, *P* = 0.028).

### Propensity to feed on honey or blood

**Fig. 5:**
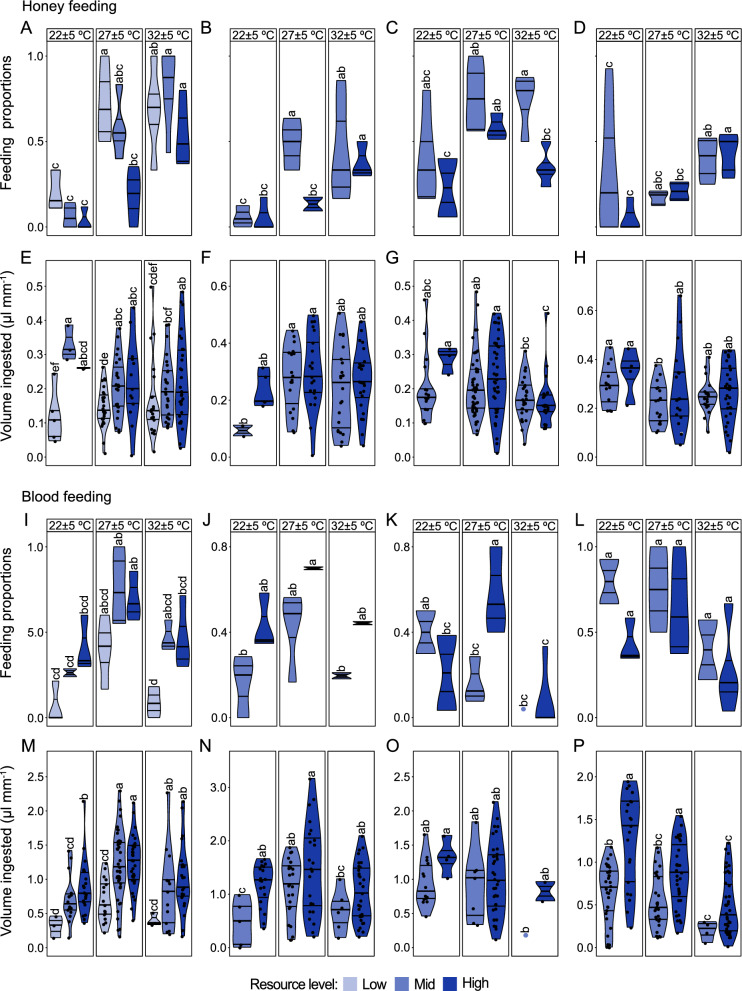
Temperature and resource level interact to influence the feeding propensity of teneral mosquitoes. Proportion of teneral female feeding on either a carbohydrate (honey) **A** *Ae. aegypti* (22 ± 5 °C: high resource *n* = 36; mid resource *n* = 35; low resource *n* = 46; 27 ± 5 °C: high resource *n* = 61; mid resource *n* = 40; low resource *n* = 36; 32 ± 5 °C: high resource *n* = 72; mid resource *n* = 39; low resource *n* = 37), **B** *An. stephensi* (22 ± 5 °C: high resource *n* = 54; mid resource *n* = 46; 27 ± 5 °C: high resource *n* = 55; mid resource *n* = 46; 32 ± 5 °C: high resource *n* = 52; mid resource *n* = 75), **C** *An. coluzzii* (22 ± 5 °C: high resource *n* = 32; mid resource *n* = 59; 27 ± 5 °C: high resource *n* = 76; mid resource *n* = 53; 32 ± 5 °C: high resource *n* = 49; mid resource *n* = 35) and **D** *An. arabiensis* (22 ± 5 °C: high resource *n* = 32; mid resource *n* = 54; 27 ± 5 °C: high resource *n* = 52; mid resource *n* = 63; 32 ± 5 °C: high resource *n* = 51; mid resource *n* = 40) or proteinaceous (blood) meal **I** *Ae. aegypti* (22 ± 5 °C: high resource *n* = 37; mid resource *n* = 32; low resource *n* = 30; 27 ± 5 °C: high resource *n* = 49; mid resource *n* = 89; low resource *n* = 37; 32 ± 5 °C: high resource *n* = 67; mid resource *n* = 30; low resource *n* = 36), **J** *An. stephensi* (22 ± 5 °C: high resource *n* = 46; mid resource *n* = 44; 27 ± 5 °C: high resource *n* = 30; mid resource *n* = 60; 32 ± 5 °C: high resource *n* = 43; mid resource *n* = 30), **K** *An. coluzzii* (22 ± 5 °C: high resource *n* = 42; mid resource *n* = 32; 27 ± 5 °C: high resource *n* = 57; mid resource *n* = 30; 32 ± 5 °C: high resource *n* = 53; mid resource *n* = 25) and **L** *An. arabiensis* (22 ± 5 °C: high resource *n* = 30; mid resource *n* = 39; 27 ± 5 °C: high resource *n* = 36; mid resource *n* = 40; 32 ± 5 °C: high resource *n* = 105; mid resource *n* = 16). Lowercase letters, from pair-wise comparisons of emmeans, indicate significant interactions between temperature and resource level on honey and blood feeding. Bars followed by different letters within the same box (between resource levels and same temperature) and bars across boxes (between temperature and same resource) are statistically different when represented by different letters (*P* < 0.05; generalised mixed-effect binomial analysis). Volume, adjusted by mean wing size, of honey ingested by teneral **E** *Ae*. *aegypti* (22 ± 5 °C: high resource *n* = 2; mid resource *n* = 3; low resource *n* = 5; 27 ± 5 °C: high resource *n* = 13; mid resource *n* = 23; low resource *n* = 27; 32 ± 5 °C: high resource *n* = 30; mid resource *n* = 27; low resource *n* = 21), **F** *An. stephensi* (22 ± 5 °C: high resource *n* = 5; mid resource *n* = 2; 27 ± 5 °C: high resource *n* = 21; mid resource *n* = 18; 32 ± 5 °C: high resource *n* = 24; mid resource *n* = 22), **G** *An. coluzzii* (22 ± 5 °C: high resource *n* = 3; mid resource *n* = 17; 27 ± 5 °C: high resource *n* = 41; mid resource *n* = 46; 32 ± 5 °C: high resource *n* = 22; mid resource *n* = 30) and **H** *An. arabiensis* (22 ± 5 °C: high resource *n* = 4; mid resource *n* = 12; 27 ± 5 °C: high resource *n* = 18; mid resource *n* = 17; *n* = ; 32 ± 5 °C: high resource *n* = 32; mid resource *n* = 20) or blood ingested by teneral female **M** *Ae*. *aegypti* (22 ± 5 °C: high resource *n* = 22; mid resource *n* = 16; low resource *n* = 3; 27 ± 5 °C: high resource *n* = 33; mid resource *n* = 45; low resource *n* = 17; 32 ± 5 °C: high resource *n* = 23; mid resource *n* = 13; low resource *n* = 3), **N** *An. stephensi* (22 ± 5 °C: high resource *n* = 19; mid resource *n* = 5; 27 ± 5 °C: high resource *n* = 24; mid resource *n* = 25; 32 ± 5 °C: high resource *n* = 31; mid resource *n* = 6), **O** *An. coluzzii* (22 ± 5 °C: high resource *n* = 4; mid resource *n* = 14; 27 ± 5 °C: high resource *n* = 36; mid resource *n* = 7; 32 ± 5 °C: high resource *n* = 2; mid resource *n* = 1) and **P** *An. arabiensis* (22 ± 5 °C: high resource *n* = 22; mid resource *n* = 32; 27 ± 5 °C: high resource *n* = 35; mid resource *n* = 28; *n* = ; 32 ± 5 °C: high resource *n* = 40; mid resource *n* = 4). Lowercase letters, from pair-wise comparisons of emmeans, indicate significant interactions between temperature and resource level on the volume ingested. Bars followed by different letters within the same box (between resource levels and same temperature) and bars across boxes (between temperature and same resource) are statistically different when represented by different letters (*P* < 0.05; Aligned Rank Transform analysis of variance). At low larval resource level *Anopheles* species did not pupate, and hence no data on feeding propensity at this resource level

The propensity of teneral females to replenish metabolic reserves with a carbohydrate meal (honey) was differentially influenced by temperature, with larval resource level negatively affecting the temperature effect in a species-dependent manner (Fig. 5A–H). While temperature and resource level during larval development did not significantly interact to influence proportion of females feeding (*Ae. aegypti*; df = 4, *χ*^2^ = 4.68, *P* = 0.32; *An. coluzzii*; df = 2, *χ*^2^ = 1.59, *P* = 0.45 and *An. arabiensis*; df = 2, *χ*^2^ = 3.20, *P* = 0.20; Fig. 5A, C, D), a higher proportion of *Ae. aegypti* and *An. coluzzii* fed as temperature increased (*Ae. aegypti*: df = 2, *χ*^2^ = 41.92, *P* < 0.001; *An. coluzzii*: df = 2, *χ*^2^ = 13.01, *P* < 0.01) an effect accentuated by restricted larval resource level (*Ae. aegypti*: df = 2, *χ*^2^ = 12.10, *P* < 0.01; *An. coluzzii*: df = 1, *χ*^2^ = 15.33, *P* < 0.001) (Fig. 5A, C). In contrast, temperature (df = 2, *χ*^2^ = 21.56, *P* < 0.001), but not resource level (df = 1, *χ*^2^ = 0.18, *P* = 0.20) enhanced the proclivity of *An. arabiensis* to feed on honey (Fig. 5D). Honey feeding by *An. stephensi* increased at high temperature when food resource level was restricted (df = 2, *χ*^2^ = 12.60, *P* < 0.01; Fig. 5B). Resource level and temperature significantly influenced the level of feeding by *Ae. aegypti* (Fig. 5E) and *An. coluzzii* (Fig. 5G), respectively (Additional file 2: Supplementary Table S2), in which *Ae. aegypti* females from high resource level fed more, whereas the level of feeding by *An. coluzzii* decreased at high temperature and resource level. The volume of honey ingested by *An. stephensi* (Fig. 5F) and *An. arabiensis* (Fig. 5H), on the other hand, was not affected by the two environmental factors (Additional file 2: Supplementary Table S2).

Temperature and larval resource level significantly interacted to influence the proclivity of teneral *An. coluzzii* (df = 2, *χ*^2^ = 18.88, *P* < 0.001; Fig. 5K) but not *Ae. aegypti* (df = 4, *χ*^2^ = 3.21, *P* = 0.52; Fig. 5I) and *An. stephensi* (df = 2, *χ*^2^ = 0.076, *P* = 0.96; Fig. 5J) to feed on a proteinaceous meal (blood), although feeding by the three species increased significantly at intermediate temperature compared with low and high temperature regimes, an effect accentuated by higher larval resource levels.

The effect of temperature and resource regimes during larval development influenced the volume of blood ingested as reflected by the proportion of *Ae. aegypti*, *An. stephensi* and *An. coluzzii* that fed. Temperature and larval resource level did not interact to influence the amount engorged by *An. stephensi* (F_2,104_ = 0.99, *P* = 0.37), *Ae. aegypti* (F_4,166_ = 0.27, *P* = 0.90) and *An. coluzzii* (F_2,58_ = 0.59, *P* = 0.56). In female *Ae. aegypti* (Fig. 5M) and *An. stephensi* (Fig. 5N), the volume was positively correlated with temperature and resource level while a similar trend of resource, but not temperature, was observed for *An. coluzzii* (Fig. 5O). The propensity of *An. arabiensis* (Fig. 5L) to feed on blood appears inversely proportionate to temperature, irrespective of the resource level during larval development, although no significant effect of the interaction of the two factors was observed (F_2,12_ = 45.40, *P* = 0.88). Moreover, while temperature and resource regimes during larval development did not significantly interact to influence the level of feeding (F_2,154_ = 1.29, *P* = 0.28), the amount ingested decreased with an increase in temperature, an effect accentuated by larval resource restriction (Fig. 5P).

## Discussion

Fluctuating diurnal temperature modulated mosquito life history traits, an effect accentuated by food availability during larval development, supporting previous studies performed under constant temperature [19, 30]. However, the response of the aquatic stages to the two environmental factors is taxon-dependent, resulting in differential carry-over effects in the adults, reflected in differential metabolic reserves and propensity to feed, as demonstrated in the current study. This may have significant and differential consequences for population dynamics and thus the vectorial capacity of mosquitoes.

Aquatic stages of mosquitoes reared at high temperatures, have a shorter developmental time likely owing to accelerated physiological processes, such as metabolic rates leading to a faster progression through the aquatic stages [4, 23, 24, 60–62]. The availability of food accentuates the observed temperature effects on time to adult emergence [19, 30, 62, 66]. Resource restriction during larval development significantly increased the time to adult emergence, while abolishing adult *Anopheles* emergence. This is likely owing to the requirement for the larvae to extend their feeding period to accumulate sufficient reserves to complete pupation [67]. The reason that *Ae. aegypti* is more tolerant to food restriction compared with the anopheline species is likely due to a better capacity to amass metabolic reserves under elevated temperature conditions, as well as differences in nutritional requirements between genera [36, 37, 52, 68, 85], which translates into differential carry-over effects in the adults.

The carry-over effects of temperature stress during the aquatic stages were evidenced by reduced adult size and survival [24, 29, 61, 62], in line with the “size and fitness rule” [4, 69], which states that bigger is better. This effect was accentuated by decreasing food resource quantity during the larval stages [19, 30]. When survival under temperature stress is independent of food stress during the non-reproductive state, *i.e.*, the larval stage in the case of this study, the carry-over effects to the reproductive state, affecting fitness, may produce a stabilising effect on populations over time [71]. These mosquitoes are likely to survive as adults largely independently of food restriction during the aquatic stages. Furthermore, behavioural adaptation may allow mosquitoes to escape high temperatures. For instance, *An. coluzzii* aestivate during hot and dry months [72] and *Ae. aegypti* seek refuges in cool human habitats [2, 73, 74].

The teneral reserves, potentially regulating the survival of adults, were differentially affected by prior temperature stress and accentuated by resource restriction during the aquatic stage. Protein content generally reflects the structural biomass of mosquitoes, *i.e*., size; however, protein can also be used as a metabolic fuel during starvation [36, 37] and abiotic stress [75]. As the protein content was normalised for the size of the female in the current study, the effects of the environmental stressors, particularly food abundance, were relatively minor within species. The genus-dependent differences in the amount of protein carried-over into the adult stage relate to disparities between *Ae. aegypti* and *Anopheles* species accumulation and utilisation of protein from the teneral reserves. *Aedes aegypti* and *Anopheles* species accumulate protein during larval development, rendering them either ‘obese’ [68] or ‘undernourished’ [36], respectively, which likely restricts the eclosion of *Anopheles* at low-level diet conditions, as also reflected in the current study. In response to stress, particularly food abundance, teneral protein content is differentially affected, with *Ae. aegypti* being able to mobilise three times less of their protein reserves than *Anopheles* [36, 37]. This correlates with a shorter life span for *Ae. aegypti* under stress.

While mosquitoes utilise protein for energy under stress, the primary metabolites relied on for energy are lipids and glycogen [36, 37]. A high content of the two long-term reserves may increase adult longevity and starvation resilience [59, 76, 77]. Survival directly correlates with adult size, with the correlation weakening with increasing temperature across all species [4, 19, 78]. At the highest temperatures, the *Anopheles* species continue to increase in size, if provided ample food as larvae, but no longer increase in adult longevity [4], indicating that the similar levels of teneral reserves across species are independent of stress conditions. This is reminiscent of the ‘energy limited tolerance to stress’ concept, in which more energy is needed for the basal maintenance of large insects to cope with temperature stress’ [79] and the need to allocate energy reserves for reproduction [4, 19, 78]. To alleviate any shortage in teneral reserves, adult females engage in feeding on different nutritional sources.

Females fed differentially on carbohydrate- and protein-rich meals, emphasising species-dependent strategies in replenishing metabolic reserves, in response to temperature- and resource-induced stress. In contrast to other insects studied, which regulate the carbon:nitrogen balance through quantities ingested [80, 81], mosquitoes separate the carbon-rich meal from the nitrogen-rich meal spatially and temporally depending on their physiological status [70], thus expanding the regulation from amount ingested to the proportion of individuals actively engaged in feeding on either resource. In response to an increase in temperature and resource restriction, females of all species had a high propensity to feed on honey demonstrating a reliance on sugar sources in response to stress. The propensity of teneral females to feed on blood was independent of temperature and resource level during larval development, whereas the volume of blood ingested was dependent on species as well as temperature and resource level during larval development. The volume of blood engorged by the teneral female is inversely proportional to the carbohydrate reserves, and regulated by temperature and resource level during larval development, indicating that while mosquitoes separate their carbohydrate- and protein-rich meals, the females appear to follow the canonical balancing model for carbon and nitrogen [70]. Taken together, female mosquitoes appear to engage in species-dependent feeding to replenish and balance metabolic reserves in response to abiotic stress.

## Conclusions

This study underscores the effects of temperature stress and diet limitation on species-dependent life history traits of aquatic and terrestrial stages of mosquitoes. A shorter development time at high temperature, independent of larval food resources, may increase population growth rate and stability, which may increase disease transmission [39, 69]. Disease transmission dynamics is, furthermore, likely to be influenced by species-dependent strategies and capacities to store metabolic reserves in response to biotic and abiotic stress [29, 36], which has ramifications for teneral adult feeding behaviour. Abiotic and biotic stress may both increase or decrease the avidity of females to blood feed, which may affect the human biting rate and hence, disease transmission [12, 35, 39, 82–84]. The comparative assessment of how different mosquito species respond to abiotic stress provides an insight to how vector fitness is affected, and needs to be incorporated into mechanistic prediction models to provide a holistic understanding of mosquito-borne disease transmission dynamics in response to climate change. We acknowledge that species used in this study originated from long standing laboratory colonies; therefore, their response to abiotic stress might differ from that of field populations. In addition, resource levels used in this study were not varied per larval instar, and hence larvae overfeeding at high resource level [85] may confound the observed effects of abiotic stress on different life history parameters, and this requires further investigation.

## Supplementary Information

Additional file 1: Supplementary Figure S1. A schematic representation of experimental set up on the effect of temperature and larval resource level on (A) immature and adult life history traits, (B) teneral metabolic reserves and (C) feeding propensity of teneral adults. Supplementary Figure S2. Schematic representation of diurnal temperature fluctuation in the experimental chambers during the experimental photoperiod. White and shaded areas represent photophase and scotophase, respectively. ZT: Zeitgeber time. Temperature increases gradually to a maximum during photophase and decreases to a minimum during scotophase. The low temperature fluctuates at a range of 17-27 °C around a mean of 22 °C, intermediate temperature at 22-32 °C around a mean of 27 °C and high temperature at 27-37 °C around a mean of 32 °C. Supplementary Figure S3. Correlation between survival and size of (A) Ae. aegypti, (B) An. stephensi, (C) An. coluzzii and (D) An. arabiensis in response to diurnal fluctuating temperature and larval resource level. R2 represents the Pearson’s correlation coefficient. Error bars are constructed using 95% confidence interval (CI) to indicate variation of the mean survival duration. The grey shading indicates the 95% CI margin.

Additional file 2: Supplementary Table S1. Pairwise linear regression analyses following the findings of significant overall models of the effect of size on survival of each mosquito species at different temperature regimes (P<0.05). Supplementary Table S2. Aligned Rank Transform analysis of variance on temperature, resource level, and their interaction on the volume of honey (carbohydrate meal) ingested by each teneral mosquito species (P<0.05).

## Data Availability

All data can be found in the Github repository: https://github.com/JuliahJacob/Effect-of-abiotic-stress-on-the-life-history-and-behaviour-of-diseasetransmitting-mosquitoes.
