# Supplementary material for: Diurnal fluctuating temperature and larval resource level interact to influence the life history and behaviour of disease-transmitting mosquitoes
Source: Parasit Vectors. 2026 Feb 21;19:150. doi: 10.1186/s13071-026-07313-4 (PMC13063854; doi:10.1186/s13071-026-07313-4)
Supplement: Supplementary file 1 — Additional file 1: Supplementary Figure S1. A schematic representation of experimental set up on the effect of temperature and larval resource level on (A) immature and adult life history traits, (B) teneral metabolic reserves and (C) feeding propensity of teneral adults. Supplementary Figure S2. Schematic representation of diurnal temperature fluctuation in the experimental chambers during the experimental photoperiod. White and shaded areas represent photophase and scotophase, respectively. ZT: Zeitgeber time. Temperature increases gradually to a maximum during photophase and decreases to a minimum during scotophase. The low temperature fluctuates at a range of 17-27 °C around a mean of 22 °C, intermediate temperature at 22-32 °C around a mean of 27 °C and high temperature at 27-37 °C around a mean of 32 °C. Supplementary Figure S3. Correlation between survival and size of (A) Ae. aegypti, (B) An. stephensi, (C) An. coluzzii and (D) An. arabiensis in response to diurnal fluctuating temperature and larval resource level. R2 represents the Pearson’s correlation coefficient. Error bars are constructed using 95% confidence interval (CI) to indicate variation of the mean survival duration. The grey shading indicates the 95% CI margin. [file 13071_2026_7313_MOESM1_ESM.pdf]

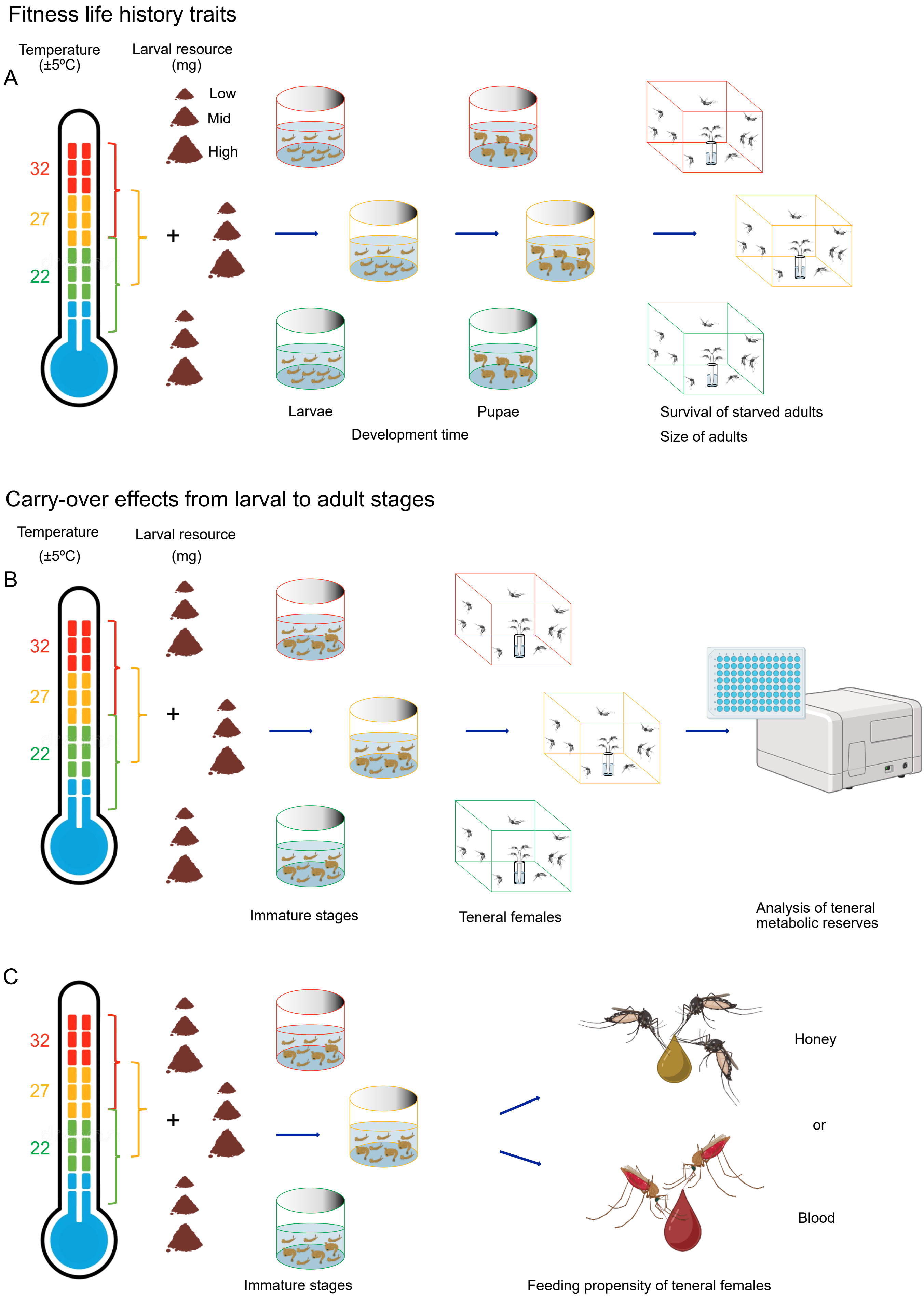

Fitness life history traits
Temperature (±5ºC)
Larval resource (mg)
A
Low
Mid
High
32
27
22
+
Larvae
Pupae
Development time
Survival of starved adults
Size of adults
Carry-over effects from larval to adult stages
Temperature (±5ºC)
Larval resource (mg)
B
32
27
22
+
Immature stages
Teneral females
Analysis of teneral metabolic reserves
C
32
27
22
+
Immature stages
Honey
or
Blood
Feeding propensity of teneral females

Figure S1. A schematic representation of experimental set up on the effect of temperature and larval resource level on (A) immature and adult life history traits, (B) teneral metabolic reserves and (C) feeding propensity of teneral adults.

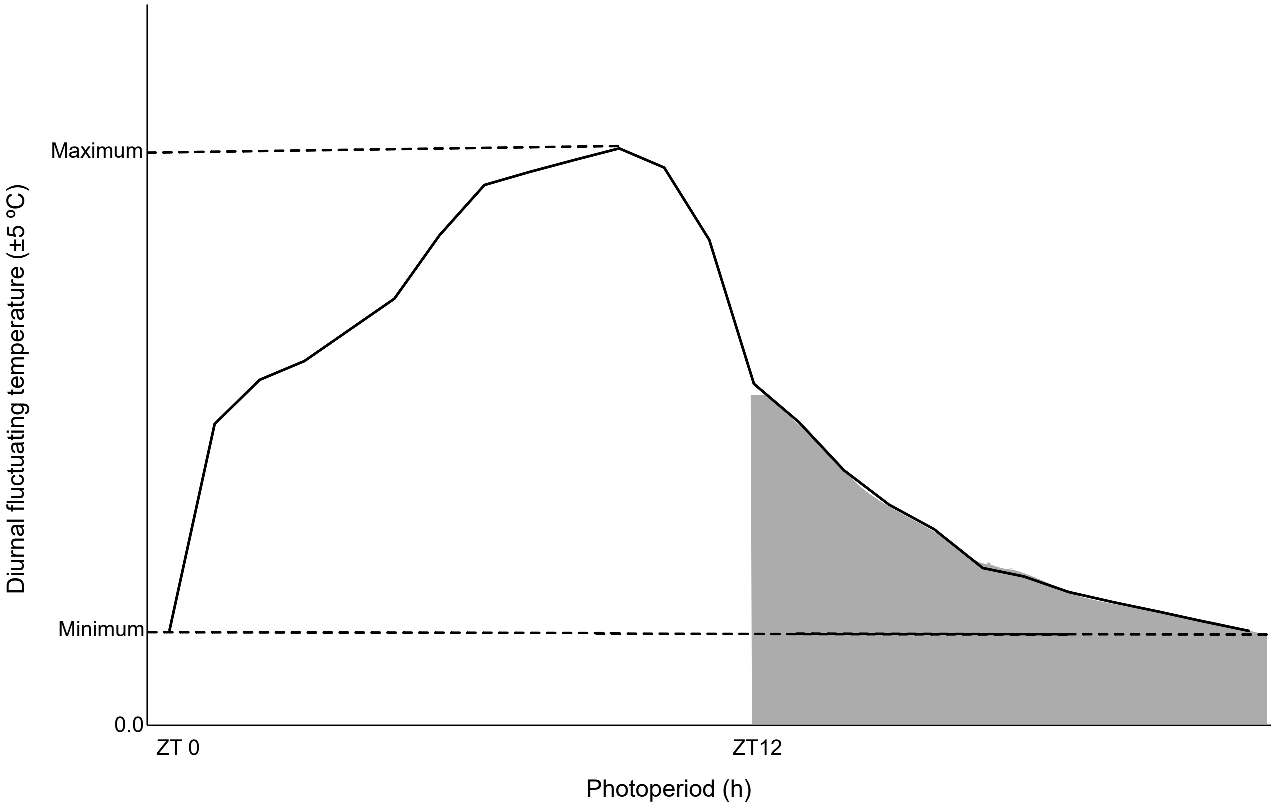

Figure S2. Schematic representation of diurnal temperature fluctuation in the experimental chambers during the experimental photoperiod. White and shaded areas represent photophase and scotophase, respectively. ZT: Zeitgeber time. Temperature increases gradually to a maximum during photophase and decreases to a minimum during scotophase. The low temperature fluctuates at a range of 17-27 °C around a mean of 22 °C, intermediate temperature at 22-32 °C around a mean of 27 °C and high temperature at 27-37 °C around a mean of 32 °C.

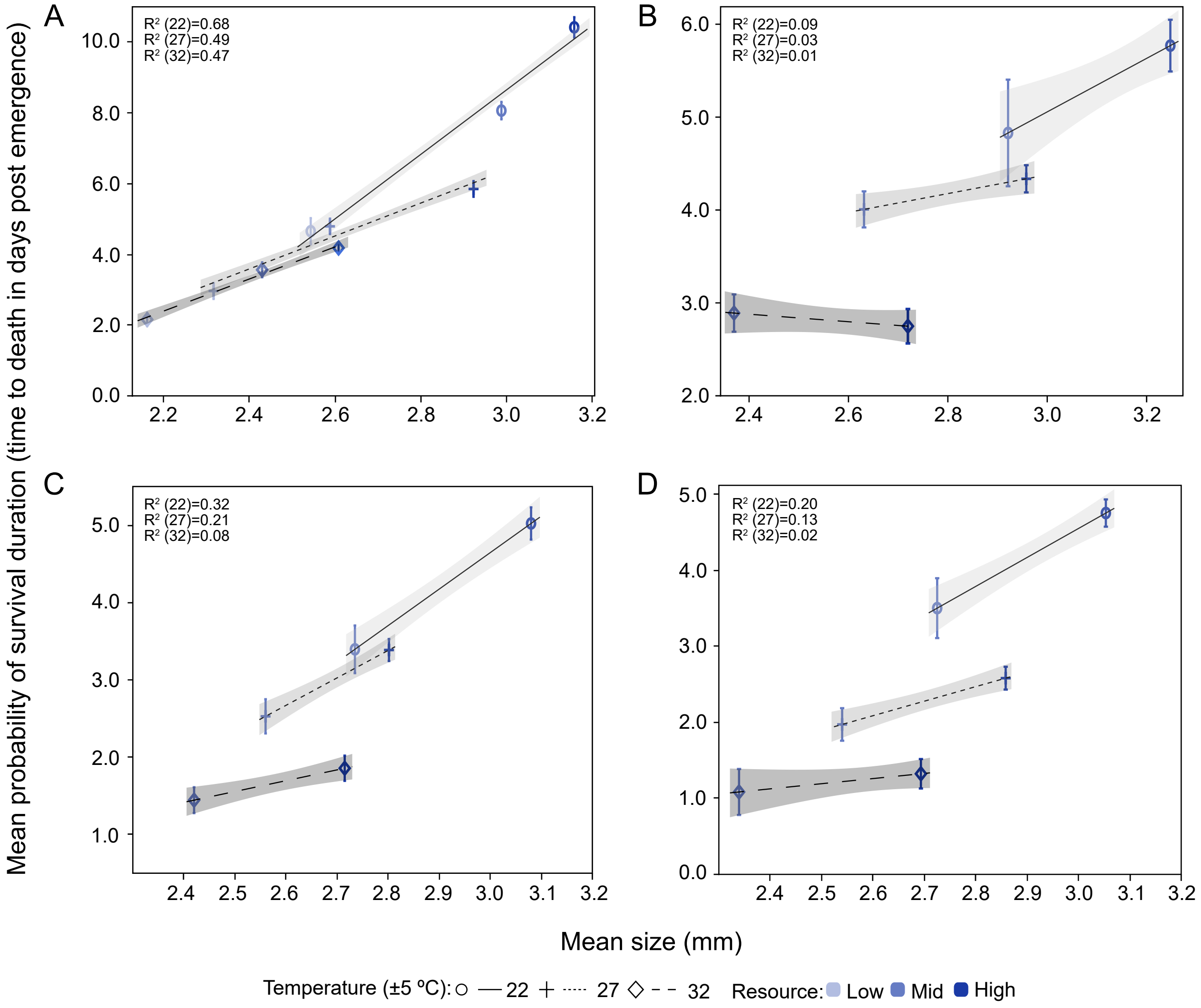

Figure S3. Correlation between survival and size of (A) *Ae. aegypti*, (B) *An. stephensi,* (C) *An. coluzzii* and (D) A*n. arabiensis* in response to diurnal fluctuating temperature and resource level. R^2 represents the Pearson's correlation coefficient. Error bars are constructed using 95% confidence interval (CI) to indicate variation of the mean survival duration. The grey shading indicates the 95% CI margin.
