# Supplementary material for: Diurnal fluctuating temperature and larval resource level interact to influence the life history and behaviour of disease-transmitting mosquitoes
Source: Parasit Vectors. 2026 Feb 21;19:150. doi: 10.1186/s13071-026-07313-4 (PMC13063854; doi:10.1186/s13071-026-07313-4)
Supplement: Supplementary file 2 — Additional file 2: Supplementary Table S1. Pairwise linear regression analyses following the findings of significant overall models of the effect of size on survival of each mosquito species at different temperature regimes (P<0.05). Supplementary Table S2. Aligned Rank Transform analysis of variance on temperature, resource level, and their interaction on the volume of honey (carbohydrate meal) ingested by each teneral mosquito species (P<0.05). [file 13071_2026_7313_MOESM2_ESM.docx]

**Table S1**. Pairwise linear regression analyses following the findings of significant overall models of the effect of size on survival of each mosquito species at different temperature regimes (P<0.05).

| Species | Temperature (±5 °C) | df_n_ | df_d_ | F value | P value |
| --- | --- | --- | --- | --- | --- |
| Aedes aegypti | 22 vs 27 | 1 | 589 | 94.12 | <0.0001 |
|  | 22 vs 32 | 1 | 569 | 75.65 | <0.0001 |
|  | 27 vs 32 | 1 | 554 | 0.083 | 0.77 |
|  |  |  |  |  |  |
| Anopheles stephensi | 22 vs 27 | 1 | 340 | 5.42 | 0.021 |
|  | 22 vs 32 | 1 | 301 | 15.05 | 0.0001 |
|  | 27 vs 32 | 1 | 407 | 6.69 | 0.01 |
|  |  |  |  |  |  |
| Anopheles coluzzii | 22 vs 27 | 1 | 342 | 2.16 | 0.14 |
|  | 22 vs 32 | 1 | 298 | 18.95 | <0.0001 |
|  | 27 vs 32 | 1 | 298 | 10.10 | 0.0016 |
|  |  |  |  |  |  |
| Anopheles arabiensis | 22 vs 27 | 1 | 338 | 6.79 | 0.0096 |
|  | 22 vs 32 | 1 | 278 | 14.08 | 0.0002 |
|  | 27 vs 32 | 1 | 250 | 4.15 | 0.043 |

**Table S2**. Aligned Rank Transform analysis of variance on temperature, resource level, and their interaction on the volume of honey (carbohydrate meal) ingested by each teneral mosquito species (P<0.05).

| Factor | Species | df_n_ | df_d_ | F value | P value |
| --- | --- | --- | --- | --- | --- |
| Temperature | *Ae. aegypti* | 2 | 142 | 0.55 | 0.56 |
| Resource |  | 2 | 142 | 5.39 | 0.0055 |
| Temp×Resource |  | 4 | 142 | 1.55 | 0.19 |
|  |  |  |  |  |  |
| Temperature | *An. stephensi* | 2 | 86 | 2.21 | 0.12 |
| Resource |  | 1 | 86 | 0.53 | 0.47 |
| Temp×Resource |  | 2 | 86 | 0.71 | 0.49 |
|  |  |  |  |  |  |
| Temperature | *An. coluzzii* | 2 | 153 | 6.30 | 0.002 |
| Resource |  | 1 | 153 | 153 | 0.19 |
| Temp×Resource |  | 2 | 153 | 3.37 | 0.037 |
|  |  |  |  |  |  |
| Temperature | *An. arabiensis* | 2 | 97 | 2.03 | 0.14 |
| Resource |  | 1 | 97 | 0.59 | 0.45 |
| Temp×Resource |  | 2 | 97 | 0.18 | 0.84 |
